# Biomimetic Dispersive Solid-Phase Microextraction: A Novel Concept for High-Throughput Estimation of Human Oral Absorption of Organic Compounds

**DOI:** 10.1021/acs.analchem.3c01749

**Published:** 2023-08-24

**Authors:** Maria Pau García-Moll, Llucia García-Moll, Enrique Javier Carrasco-Correa, Miquel Oliver, Ernesto Francisco Simó-Alfonso, Manuel Miró

**Affiliations:** † FI-TRACE Group, Department of Chemistry, University of the Balearic Islands, Carretera de Valldemossa, km 7.5, Palma de Mallorca E-07122, Spain; ‡ CLECEM Group, Department of Analytical Chemistry, University of Valencia, C/Doctor Moliner, 50, Burjassot, Valencia 46100, Spain

## Abstract

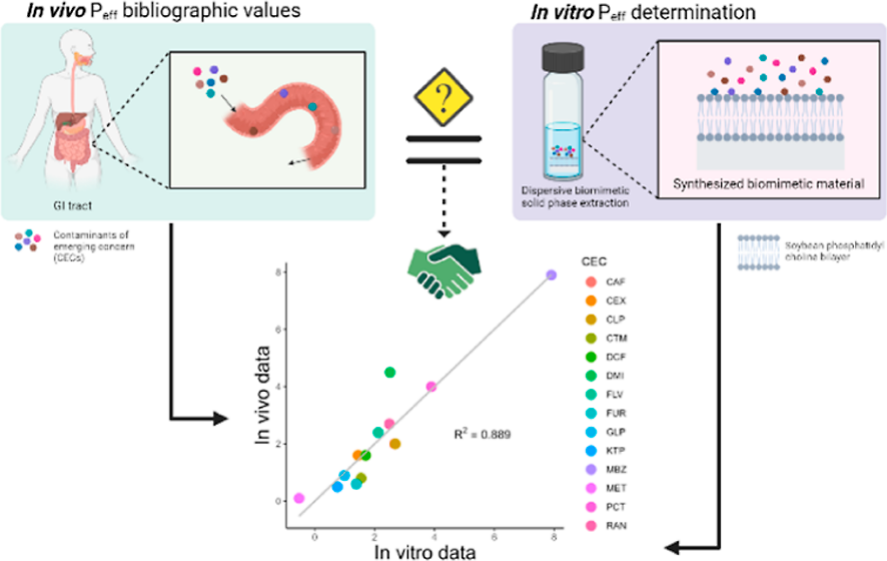

There is a quest for a novel in vitro analytical methodology that is properly validated for the prediction of human oral absorption and bioaccumulation of organic compounds with no need of animal models. The traditional log *P* parameter might not serve to predict bioparameters accurately inasmuch as it merely accounts for the hydrophobicity of the compound, but the actual interaction with the components of eukaryotic cells is neglected. This contribution proposes for the first time a novel biomimetic microextraction approach capitalized on immobilized phosphatidylcholine as a plasma membrane surrogate onto organic polymeric sorptive phases for the estimation of human intestinal effective permeability of a number of pharmaceuticals that are also deemed contaminants of emerging concern in environmental settings. A comprehensive exploration of the conformation of the lipid structure onto the surfaces is undertaken so as to discriminate the generation of either lipid monolayers or bilayers or the attachment of lipid nanovesicles. The experimentally obtained biomimetic extraction data is proven to be a superb parameter against other molecular descriptors for the development of reliable prediction models of human jejunum permeability with *R*^2^ = 0.76, but the incorporation of log *D* and the number of aromatic rings in multiple linear regression equations enabled improved correlations up to *R*^2^ = 0.88. This work is expected to open new avenues for expeditious in vitro screening methods for oral absorption of organic contaminants of emerging concern in human exposomics.

Human oral absorption (HOA) refers to the absorption-related processes that a target compound undergoes throughout the gastrointestinal tract (GIT)^1^ and depends most likely on the molecule size, hydrogen bonding interactions, and overall lipophilicity, but also on the shape and chemical conformations of the target as well.^2,3^ In particular, the human intestinal effective permeability (*P*_eff_) values of target compounds are of great importance in HOA to understand their partitioning across the GIT as demanded, e.g., in pharmacokinetics, drug design, and toxicological studies. Focus should be given to the *P*_eff_ assessment throughout the small intestine (duodenum, jejunum, and ileum), which embraces the main sites for GIT absorption,^4^ and specially on the jejunum (ca. half of the total length of the small intestine).^5^

To evaluate the HOA of compounds across the GIT, in vivo methods have been proposed, characterized, and standardized over the past decades.^6,7^ Notwithstanding, in vivo tests must be carried out over prolonged periods of time to ensure that the target compound is absorbed, distributed, excreted, and, in some cases, metabolized and thus do not bear high-throughput credentials. In addition, in vivo (toxicity) assays that require specialized staff are performed with animal models, with the subsequent generation of ethical controversy. Also, they might not be sensitive enough to evaluate deleterious effects at environmentally relevant concentrations of pollutants.^8^ To this end, regulatory entities, such as the European Union’s Chemical Registration, Evaluation, Authorization and Restriction Program (REACH) suggested replacing in vivo assays with their in vitro counterparts as appealing, cost-effective, and functional alternative tools without the need of animal models,^9^ in line with white analytical chemistry principles.^10^ In vitro testing assays are faster, more reproducible, do not raise ethical concerns, and enable experimental estimation of the *P*_eff_ to serve as a conservative scenario of the maximum human bioavailability on account of the ability of the target species to cross biological/intestinal membranes. Therefore, in vitro methodologies based on passive diffusion using modified membrane surrogates, such as (i) the parallel artificial permeability assay, (ii) the phospholipid vesicle-based permeation assay, and (iii) the artificial membrane insert, have been proposed in the past years to evaluate *P*_eff_.^11^ However, human permeability is not governed only by passive diffusion, and thus, dynamic permeation models, mainly focused on liquid chromatographic techniques, have taken the lead.^12^ Dynamic methods capitalizing on phosphatidylcholine (PC) or other phospholipid derivatives, cholesterol, and/or plasma components mimic closely the composition of plasma membranes of eukaryotic cells^13^ and are able to simulate the interaction of targets with cell membranes under changing conditions. The three main chromatographic techniques that have been adopted as artificial biomimetic membrane models using cell-free membrane surrogates are (i) immobilized artificial membrane chromatography (IAM),^11^ (ii) biopartitioning micellar chromatography (BM),^14^ and (iii) immobilized plasma protein chromatography (IPP).^15^ Nevertheless, IAM, BM, and IPP chromatography have been accepted by many practitioners;^16–19^ all of the above separation approaches are tedious and time-consuming and are unable, whenever coupled to UV–vis detection methods, to detect several compounds simultaneously because their resolution is poor. In addition, they might introduce other unspecific (bio)interactions that ward off the prediction models.

Biomimetic sorptive microextraction approaches, on account of the wide gamut of phases and extraction modes available, viz., dispersive, magnetic, packed-bed, pipet-tip, and spin column, to name just a few,^20,21^ might be regarded as excellent alternatives to passive diffusion and dynamic partitioning modes for in vitro *P*_eff_ prediction. Dispersive solid-phase extraction (dSPE) bears some unique features, such as the fast attainment of steady-state extraction conditions and the simplicity of the operational procedures. In brief, a solid material with (bio)chemical moieties is in dSPE agitated with the sample for efficient trapping of the target species while removing the liquid sample by centrifugation or filtration.^20^ In this context, it is important to note that porous organic polymers (POPs) have attracted a great deal of attention as sorbent materials in SPE because of their wide pH-range stability, facile synthesis, and simple protocols for (bio)chemical modification to ameliorate the polymer’s surface area while triggering specific molecular interactions with the targets. For example, research efforts were geared toward the combination of PC^22^ or PC derivatives^23,24^ with POPs in separation methods, yet to the best of our knowledge, biomimetic PC-laden POPs have not been proposed for the prediction of HOA-related bioparameters as yet.

In this work, large unilamellar vesicles (LUVs) are exploited as a PC source to endow glycidyl methacrylate (GMA)-based POPs with biomimetic features of biological lipid membranes. Hence, a novel in vitro physiologically relevant extraction approach capitalized upon dispersive biomimetic solid-phase extraction (d-BMSPE), and a new bioparameter named relative mol bioextraction (RMBE) are herein presented for the high-throughput estimation of HOA of organic compounds. Validation of the d-BMSPE procedure was undertaken by the bioextraction of ten pharmaceuticals that are also regarded as contaminants of emerging concern (CEC).^25–34^ The amount of CEC extracted by the biosorbent was evaluated as a core parameter along with other molecular descriptors for the reliable prediction of human jejunum permeability by resorting to multiple linear regression (MLR) methods.

## Experimental Section

Description of (i) reagents and standards, (ii) analytical instrumentation, (iii) synthesis of LUVs, and (iv) chromatographic assays are available in the Supporting Information (SI). The pharmaceutical compounds herein investigated include paracetamol (PCT), ranitidine (RNT), caffeine (CAF), chloramphenicol (CLP), furosemide (FUR), mebendazole (MBZ), glipizide (GLP), ketoprofen (KTP), diclofenac (DCF), fluvastatin (FLV), desipramine (DMI), cephalexin (CEX), cimetidine (CTM), and metformin (MET).

### Synthesis of the Porous Organic Monolithic Material

#### Synthesis of the Glycidyl Methacrylate-Based Monolithic Phase

Polymer methacrylate-based monolithic phases are prepared from a polymerization mixture containing 20% wt GMA as the functional monomer, 5% wt ethylene glycol dimethacrylate (EDMA) as the cross-linker, and 5% wt 1-dodecanol and 70% wt cyclohexanol as porogens. In addition, 1% wt lauroyl peroxide (LPO) with respect to the total amount of reagents is added as the initiator.^35^ The reagent mixture is vortexed for 20 s and subjected to bath sonication for 10 min before polymerization by thermal initiation (20 h at 60 °C). The white solid obtained is washed three times with 40 mL of methanol using a vacuum pump and then dried at 60 °C overnight. Finally, the GMA-based monolith is grounded with a mortar and sieved to a particle size spanning from 63 to 250 μm.

#### Synthesis of PC-Laden POP

**Figure 1:**
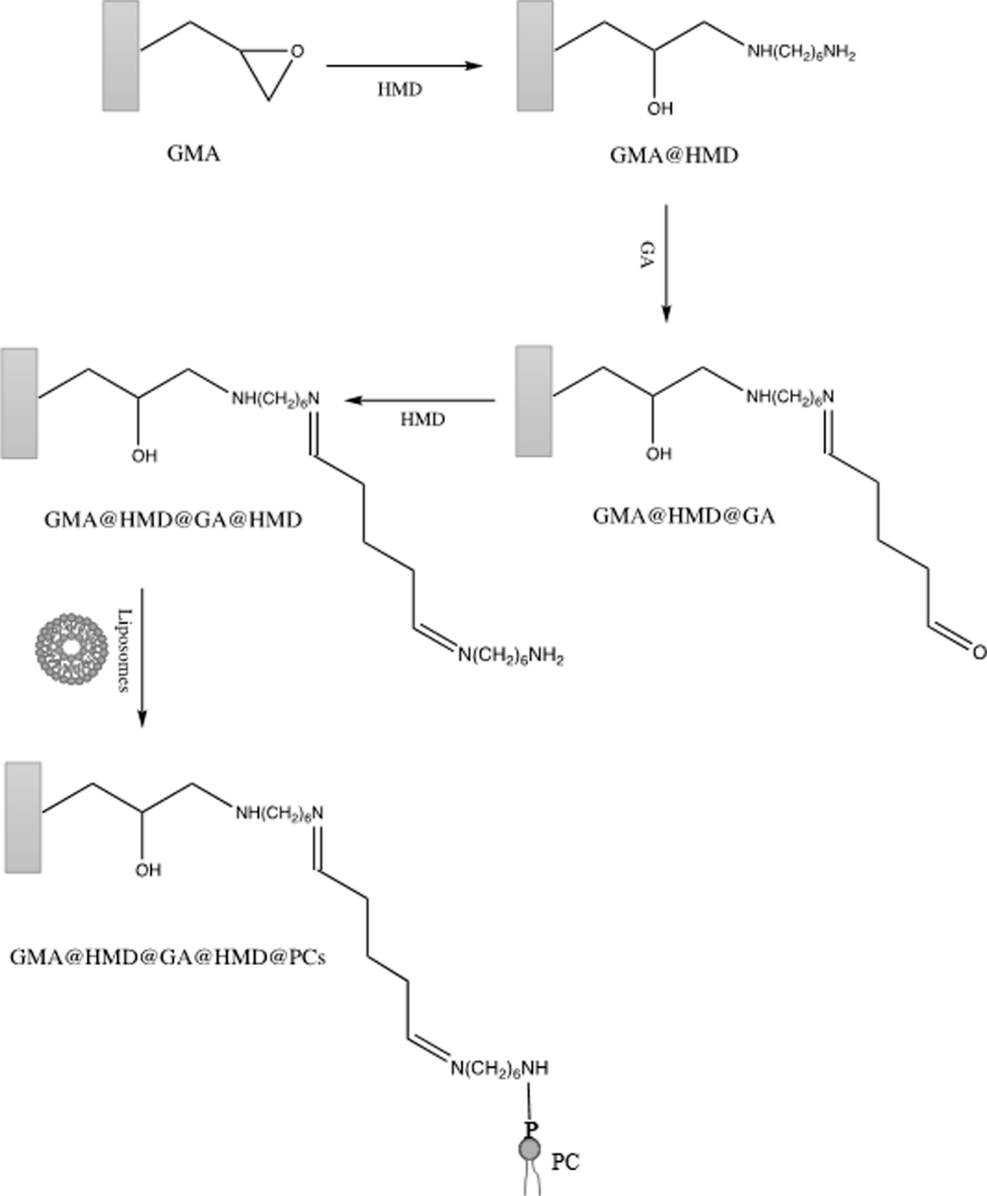
Synthesis of PC-based biomimetic sorbent using GMA-based POP.

The synthesis of the biomimetic monolith powder involves the covalent attachment of a spacer arm of 12 C to avoid steric hindrance when anchoring PC.^22^ The experimental procedure is depicted in Figure 1, and the experimental protocol for decoration of the POP is described as follows: first, 20 mL of 2 M hexamethylenediamine (HMD) solution is stirred with 1 g of the ground GMA-based monolith powder at 600 rpm for 2 h at 60 °C. The as-obtained GMA@HMD powder is cleaned with water until neutral pH, followed by rinsing with 40 mL of methanol by vacuum filtering and finally drying at 60 °C overnight. The powder is then made to react with a solution containing 1 M glutaraldehyde (GA) for 12 h at room temperature (R.T.) at 600 rpm (20 mL solution g^–1^ GMA@HMD powder). The resulting material, GMA@HMD@GA, is cleaned as per the previous step. Aiming at obtaining the 12 C spacer, another HMD moiety is attached to the GMA@HMD@GA by reaction with a 2 M HMD solution for 12 h at R.T. at 600 rpm (20 mL solution g^–1^ GMA@HMD@GA powder). The GMA@HMD@GA@HMD sorbent is then cleaned as described in the previous steps. Finally, the incorporation of PC is done by mixing GMA@HMD@GA@HMD with a previously 30 min stirred solution containing 100 mM 1-ethyl-3-(3-(dimethylamino)propyl)carbodiimide (EDC) and 20 mM LUVs (see Supporting Information) in 0.1 M imidazole (20 mL solution g^–1^ GMA@HMD@GA@HMD powder). The reaction is performed by stirring at 600 rpm for 30 min at R.T., as a result of which the biomimetic material GMA@HMD@GA@HMD@PCs is obtained. Pending use, the powder is cleaned with 50 mL of water in a vacuum system, dried at R.T., and kept in the dark.

### Quantification of PC Attached to GA-Based POPs

The amount of the attached PC to the monolithic phase is calculated following the scheme shown in Figure S1. For this purpose, a 2 mL solution (so-called PC_i_) of 20 mM LUVs, containing 2.5 mM PBS, 100 mM EDC, and 0.1 M imidazole is mixed with 100 mg of GMA@HMD@GA@HMD, followed by the reaction procedure previously described to attach PC (see above). A blank solution without LUVs is also mixed with the POP powder. The solutions are then centrifuged to separate the solid material and the supernatant (so-called PC_f_). At this moment, the linked PC onto the surface can be quantified by the Stewart’s method (SM).^36^ Briefly, 3 μL of the liquid supernatant (PC_f_) of the sample or blank is mixed with 0.5 mL of 0.1 M iron(III) thiocyanate (see Supporting Information) and 1 mL of chloroform and vortexed for 1 min. Then, 700 μL of the chloroform phase that extracts PC containing the iron(III) thiocyanate complex by reversed micelle formation is collected for further spectrophotometric analysis. The total amount of PC bound to the monolith (PC_M_) is measured by eq 1.
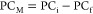
1where PC_M_ is the PC linked to the monolith, PC_i_ is the initial amount of PC in the reaction medium, and PC_f_ is the surplus of PC that is not bound to the monolith, all of them in mmol PC g^–1^ monolith.

In addition, the covalently (PC_Mca_) and the noncovalently (PC_Mnca_) attached PC, but both linked to the monolith surface, are measured by the following procedure: the final solid material (GMA@HMD@GA@HMD@PCs) and its blank counterpart (without PC) are cleaned 5 times with 40 mL of water, and then, mixed with 0.5 mL of 0.1 M of iron(III) thiocyanate and 1 mL of chloroform (SM) that dissolves PCM_nca_ from the sorbent. After vortexing for ca. 1 min, the suspensions are centrifuged for 1 min. Then, 400 μL of the chloroform phase is used to quantify, after appropriate dilution, the amount of PC_Mnca_. PC_Mca_ is obtained by eq 2
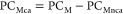
2

To evaluate the PC concentrations of the distinct phases, a calibration plot of absorbance vs [PC] in chloroform is built following SM. This calibration was also used to calculate the actual PC concentration of the LUVs synthesized. The absorbance of the first derivative of the PC–iron(III) thiocyanate supramolecular entity at 400 nm, corrected by the signal obtained by the corresponding blank (1st derivative at 400 nm), is used for all calculations.

### Dispersive Biomimetic Solid-Phase Extraction Procedure

The experimental procedure starts by spiking 5 mL of PBS at pH 7.4 mimicking pH conditions from the small intestine with 3.5 mg L^–1^ of the selected CEC and mixing with ca. 40 mg of PC-modified POP material. The material is dispersed gently in the solution at 15 rpm for 30 min at physiological temperature (37 °C) using a lab rotator, followed by separation of the biomimetic material by centrifugation. After separation, the supernatant of the biomimetic extractions is analyzed by HPLC. The bioextractable fraction of every CEC is determined by subtraction from the original spike concentration.

## Results and Discussion

### Preliminary Considerations for the Preparation of Biomimetic Polymers

The choice of a polymeric material with appropriate porosity and mechanical stability is crucial to perform any SPE modality. To ensure these requirements, a thermal polymerized GMA-based monolithic material was selected as reported elsewhere.^22^ In fact, the poly(GMA-*co*-EDMA) monolith has a large number of reactive epoxide groups susceptible to be readily functionalized and also bears good permeability.^37^ The introduction of specific biomimetic interactions is herein designed by incorporating PC molecules through water-dispersed LUVs as a source of phospholipids. For this purpose, a previously reported reaction pathway by Moravcová et al.^22^ is adopted for the attachment of PC available in the LUVs to the surface of the polymers, yet throughout the P moieties rather than the acyl chains of PC.

### Physicochemical Characterization of the Biomimetic Material

The actual conformation that the LUVs [hydrodynamic diameter (*Z*-average) of ∼120 nm and a polydispersity index (PdI) of 0.064 as obtained by dynamic light scattering] acquire after their attachment onto the surface of the monolithic structure is elucidated using a modified SM.^36^ This methodology, based on the colorimetric determination of phospholipids (see Experimental Section), enables differentiating against the distinctly different supramolecular structures that the PC can conform to on the monolithic surface, that is monolayer, bilayer, or vesicle. To this end, (i) the total bound PC fraction given as μmol per g of polymer (PC_M_), (ii) the covalently attached PC fraction in μmol per g of polymer (PC_Mca_), and (iii) the noncovalently attached PC fraction in μmol per g of polymer (PC_Mnca_) should be calculated. The PC_Mca_/PC_Mnca_ ratio aids at shedding light into the actual PC structure onto the monolithic surface. In fact, ratios >1 signaled the formation of a monolayer preferably because the amount of covalently attached PC is higher than the noncovalently attached PC. Ratios ∼1 indicate that the conformation of PC is mainly dominated by a bilayer structure. In other words, approximately half of the total bound PC is covalently attached and half is noncovalently attached. On the contrary, ratios <1 signal that the noncovalently attached PC fraction predominates on the porous polymer and, thus, vesicles (LUVs) are expected to be the most common PC structures onto the material because they will in turn bear a large amount of noncovalently attached PC. Nevertheless, these results do not ensure that the estimated structure is the only available conformation in the material but the dominant one. According to the ratios obtained by the SM, the total bound PC is 217 ± 17 μmol PC/g monolith while the covalently attached and noncovalently attached are 206 ± 20 and 11 ± 3 μmol PC/g monolith, respectively. The PC_Mca_/PC_Mnca_ ratio, in our case, is 20 ± 8, thereby signaling that the polymer, in case of a single PC structure onto the surface, is mainly decorated by a PC monolayer with an average of 200 PC molecules covalently attached for every 10 PC molecules noncovalently attached.

**Figure 2:**
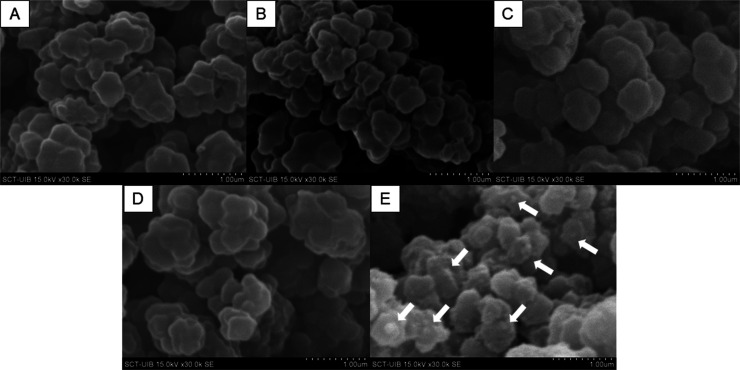
SEM micrographs at 30× magnification of the various monoliths obtained across the synthetic protocol steps: GMA (A), GMA@HMD (B), GMA@HMD@GA (C), GMA@HMD@GA@HMD (D), and GMA@HMD@GA@HMD@PCs (E). White arrows indicate the putative LUVs attached onto the surface.

In order to corroborate the PC structure onto the monolithic surface, SEM micrographs of all steps of the different reactions performed to synthesize the GMA@HMD@GA@HMD@PCs monolith were evaluated (Figure 2A–E). As can be seen in Figure 2, no significant differences on the globule size and roughness were observed in the course of the first reaction steps (Figure 2A–B), but a small increase of the globule size can be seen for the polymers modified with GA (Figure 2C) and following the subsequent reaction with HMD (Figure 2D). An apparent increase of the roughness of the characteristic globular structure of the monolith is however identified in Figure 2E, that is, GMA@HMD@GA@HMD@PCs. The miniglobules observed (white arrows in Figure 2E) onto the monolith globule surface with diameters ranging between 80 and 200 nm are most likely occasioned by directly attached LUVs. In any case, most of the surface does not have microscale globules, which is in good agreement with the SM results. This experimental finding again signaled that the main PC conformation of the biosorbent is the monolayer containing few LUVs randomly distributed over the surface.

### Preliminary Considerations for the Simulation of Permeability in the Jejunum

Following the fabrication of the biomimetic polymer, several considerations should be set before carrying out the d-BMSPE. First, the amount of PC on the modified polymer (GMA@HMD@GA@HMD@PCs) to simulate the absorption area of the jejunum needs to be evaluated. Second, a realistic concentration of the CECs should be adopted to mimic the expected concentrations in the GIT. It should be noted that previous reports on in vitro drug testing for permeation studies administered oral concentrations in the range 100–50,000 mg/L (doses between 2 and 1000 mg) for the selected targets.^6,38–40^ In our work, the doses of the pharmaceuticals were decreased down to 70 μg (3.5 mg/L in 5 mL of PBS buffer at 37 °C) in line with reliable toxicological/toxicokinetic studies of CEC in acute/chronic exposition tests down to the μg level using animal models.^41^

Aimed at estimating the amount of polymer for in vitro *P*_eff_ related extractions, the surface area of the jejunum was taken as about half of the total length of the small intestine,^5,42^ that is, 15 m^2^. Because the size of the microvilli of the human jejunal epithelial cell is ca. 0.1 μm diameter,^43^ 100 nm spherical LUVs were selected as jejunal epithelial cell surrogates to calculate the theoretical amount of PC that will cover the entire jejunum. The number of spherically shaped LUVs necessary to simulate the entire surface area of the jejunum was ca. 4.7 × 10^14^ LUVs. The PC monomers contained in the above LUVs were calculated using eq 3
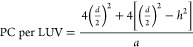
3in which 4π(*d*/2)^2^ is the surface area of the vesicle’s external monolayer, *d* is the diameter of the LUV, *h* is the thickness of the phospholipid bilayer (i.e., ≈5 nm), and *a* is the area of a single phospholipid head (ca. 1 nm^2^). Density functional theory theoretical calculations using the main fatty acid constituent of natural PC were leveraged to estimate the *h* and *a* parameters, obtaining values of 5.6 nm, and 1.3 nm^2^, respectively.^44^ Hence, the number of PC molecules per LUV calculated using eq 3 is about 48,000, and the maximum amount of PC that entirely covers the jejunum surface is ca. 37 μmol.

The estimated liquid volume under fasted conditions of the jejunum is ca. 20 mL.^45^ However, in this work, solutions of 5 mL were selected in order to reduce the amount of biomimetic material used in every extraction protocol. Therefore, only 1/4 of the total PC amount calculated above (ca. 9 μmol) should be used to maintain the PC to jejunum liquid ratio. A crucial experimental parameter in our work is the amount of PC-laden biopolymer necessary to simulate the absorption area of the jejunum. Based on the total amount of PC attached according to the SM results (217 μmol PC/g), 40 mg of GMA@HMD@GA@HMD@PC bears virtually the same number of PC molecules than the jejunum surrogate.

With respect to the remainder of experimental parameters, a gentle agitation was performed at 15 rpm to just enable a good dispersion of the polymer in the test solution. The extraction time was set to 30 min according to the average intestinal transit time and the length of the jejunum over the total length of the small intestine.^46^ Finally, the extraction temperature was set to 37 °C to simulate physiological conditions.

### Estimation of the Human Effective Permeability across the Jejunum by Dispersive Biomimetic Solid-Phase Extraction

The extraction efficiencies of (i) the free polymer (GMA), (ii) the polymer obtained prior to PC attachment (GMA@HMD@GA@HMD), and (iii) the proposed biomimetic material (GMA@HMD@GA@HMD@PC) were first evaluated using two analytes of distinct polarity (RNT and DCF with log *P* of 0.99 and 4.4, respectively). We have observed that the extraction efficiency of both analytes dropped with the incorporation of PC onto the surface of the material against those obtained with the GMA and GMA@HMD@GA@HMD counterparts. As to the GMA monolith, the extraction efficiency was around 30 and 100% for RNT and DCF, respectively. The surface of the GMA contains epoxy groups and short aliphatic chains from the methacrylate monomers, thus promoting reversed-phase interactions.^35,37^ The extraction efficiency of GMA@HMD@GA@HMD for DCF decreased down to 40% (no appreciable change for the most polar RNT analyte) because the hydrophobic moieties are now less accessible to the DCF. The notable change of the DCF extraction efficiency demonstrates the good surface coverage and thus the low availability of the parent nonbiomimetic GMA surface for the target analytes. As to the incorporation of PC, the extraction efficiencies dropped from 40 down to 10% and 30 down to 15% approximately for DCF and RNT, respectively. This observation signaled the relevance of the zwitterionic and amphiphilic PC molecules to confer entirely new biomimetic interactions with the analytes.

The absolute d-BMSPE recoveries for the 14 compounds compiled in Table 1 ranged between 0.9 (for MET) and 82.8% (for MBZ). A moderate correlation was observed by plotting d-BMSPE recoveries against in vivo *P*_eff_ data (*R*^2^ = 0.617), but one of the analytes (MBZ) was proven to distort the model because of more than 2-fold greater extraction recoveries (82.8%) than the rest of organic compounds. In fact, the determination coefficient dropped down to a mere 0.109 in the absence of MBZ. Therefore, in vitro d-BMSPE data does not suffice for *P*_eff_ prediction for targets within a broad range of polarity, and therefore additional molecular descriptors to account for all experimental variance should be incorporated in the study, with and without MBZ.

**Table 1:** Physicochemical Parameters and Molecular Descriptors for *P*_eff_ Prediction of Pharmaceuticals/CECs

CEC	d-BMSPE extraction efficiency (%)	RMBE·10^4^ (mol CEC/mol PC)	p*K*_a_^a^^,^^b^	log *P*^a^	log *D*^a^ (7.4)	aromatic ring	H-bond donor	H-bond acceptor	experimental *P*_eff_·10^4^ (cm/ s)
PCT	15.3 ± 0.8	60.3 ± 1.5	9.5 (acid group)	0.91	0.90	1	2	2	4.0^6^
RNT	16.3 ± 1.2	34 ± 3	8.1 (basic group)	0.99	0.04	1	2	5	2.7^48^
CAF	22.3 ± 0.9	70 ± 2	10.4 (basic group)	–0.55	–0.55	2	0	3	2.0^38^
CLP	18 ± 6	28.4 ± 1.6	10.9 (acid group)	0.88	0.88	1	3	5	2.0^38^
FUR	26.3 ± 1.1	49.8 ± 0.9	4.2/9.8 (acid groups)	1.75	–1.63	2	3	5	0.6^49^
MBZ	82.8 ± 0.4	184 ± 5	3.5 (basic group)	3.26	3.23	3	2	4	7.9^50^
GLP	9 ± 5	11 ± 2	5.9 (acid group)	1.43	0.54	2	3	6	0.9^38^
KTP	3 ± 2	5.7 ± 1.8	4.6 (acid group)	3.61	0.45	2	1	3	0.5^39^
DCF	10.6 ± 1.1	22.6 ± 0.8	4.5 (acid group)	4.26	1.10	2	2	3	1.6^40^
FLV	37 ± 5	58.0 ± 2.0	4.6 (acid group)	3.83	1.05	3	3	4	2.4^48^
DMI	14.1 ± 0.1	40.8 ± 0.3	2.8/10 (basic groups)	3.90	1.37	2	1	2	4.5^48^
CEX	25.6 ± 2.5	39.1 ± 2.8	3.5/11.9/12.7 (acid groups) 7.2 (basic group)	–2.14	–2.49	1	3	5	1.6^6^
CTM	4.6 ± 2.1	9.6 ± 1.7	6.5 (basic group)	–0.11	–0.22	1	3	5	0.8^51^
MET	0.9 ± 0.1	3.6 ± 0.7	10.3/12.3 (basic groups)	–0.92	–5.62	0	4	5	0.1^52^

a Obtained from chemicalize (Chemaxon Ltd.).

b Used the strongest acidic p*K*_a_ or alternatively the weakest basic p*K*_a_ in the model.

### Development and Validation of In Vitro Models to Predict Human Intestinal Permeability

Several physicochemical parameters and molecular descriptors (viz., p*K*_a_, Log *P*, Log *D* (pH 7.4), number of aromatic rings (ARs), number of H donors, and number of H acceptors) of the studied compounds (see Table 1) were investigated for reliable estimation of in vivo *P*_eff_. In addition, a new bioparameter, “RMBE” that stands for "Relative Mol BioExtraction", is herein proposed for the first time. RMBE is defined as the extraction efficiency expressed as mol of analyte extracted per mol of PC attached to the biomimetic material under physiologically simulated conditions (e.g., extraction using PBS buffer at 37 °C, or gastrointestinal fluid surrogates, or actual human fluids, among others). Taking into consideration all physicochemical parameters and molecular descriptors described above, several RMBE-based models with data obtained by d-BMSPE in PBS were tested (see Table S1), with the most representative being shown in Table 2.

**Table 2:** Non-Standardized Coefficients of the Distinct MLR Prediction Models for Estimation of the Effective Permeability of Organic Species in the Human Intestine^b^

model	constant parameter	RMBE·10^4^ (μmol CEC/mol PC)	log *P*	log *D* (7.4)	aromatic ring	H-bond acceptor	*R*^2^
1	1.71		0.363				0.1264
2	2.29			0.649			0.4329
2^a^	1.94			0.377			0.3035
**3**	**0.51**	**0.040**					**0.7628**
3^a^	0.56	0.038					0.3826
**4**	**0.82**	**0.033**		**0.288**			**0.8266**
4^a^	0.86	0.032		0.288			0.5489
**5**	**2.05**	**0.038**		**0.477**	**–0.864**		**0.8828**
5^a^	2.01	0.040		0.479	–0.874		0.6956
**14**	**3.15**	**0.038**		**0.415**	**–0.859**	**–0.272**	**0.9065**
**14**^a^	**3.40**	**0.038**		**0.400**	**–0.832**	**–0.308**	**0.7621**

a Model obtained without MBZ.

b Bold coefficients for those MLR models bearing *R*^2^ > 0.75.

Notwithstanding several authors^11^ had sought correlations of log *P* against experimental *P*_eff_ to predict HOA by merely contemplating hydrophobic interactions, the correlation in our case is negligible (*R*^2^ = 0.126, see first model in Table 2). On the other hand, by replacing log *P* with log *D* at pH = 7.4, *R*^2^ improves up to 0.435 (model 2 in Table 2), but after the removal of MBZ, *R*^2^ decreases down to 0.304 (model 2* in Table 2). By translating d-BMSPE efficiency from % to RMBE (see Table 1) and plotting against the experimental *P*_eff_ values, the correlation was acceptable (*R*^2^ = 0.763, model 3 from Table 2), but without MBZ, *R*^2^ decreased down to 0.38. These findings suggested that the use of a single parameter to predict *P*_eff_ does not suffice to obtain reliable models. Hence, other molecular descriptors (see Table 1) were incorporated in MLR equations. To this end, a preliminary multicollinearity study of the parameters selected (Table 1) was conducted to predict *P*_eff_ (Figure S2). A multicollinearity plot displays the correlation of the selected dependent parameter (*P*_eff_) against potential predictors. According to the graphic table in Figure S2, *P*_eff_ is highly correlated with RMBE but with Log *D* at pH 7.4 and the number of AR as well. Hence, two extra MLR-based models were evaluated (models 4 and 5 in Table 2). Model 4 incorporated RMBE data and the Log *D*. An excellent correlation was obtained with MBZ (*R*^2^ = 0.827) but dropped down to 0.549 without the analyte, yet the predictive capacity of the model is significantly improved as compared to the previous models for biomimetic systems in the literature.^11^ By adding the number of ARs to the MLR model (model 5), good correlations were obtained both with and without MBZ, namely *R*^2^ = 0.883 and *R*^2^ = 0.696, respectively. Additional models containing 4 predictors were tested (see Table S1), but correlation was not significantly improved, just minimally with model 14 (*R*^2^ = 0.907 with MBZ) and model 14* (*R*^2^ = 0.762 without MBZ). Therefore, model 5 with merely three descriptors was selected for further studies.

**Figure 3:**
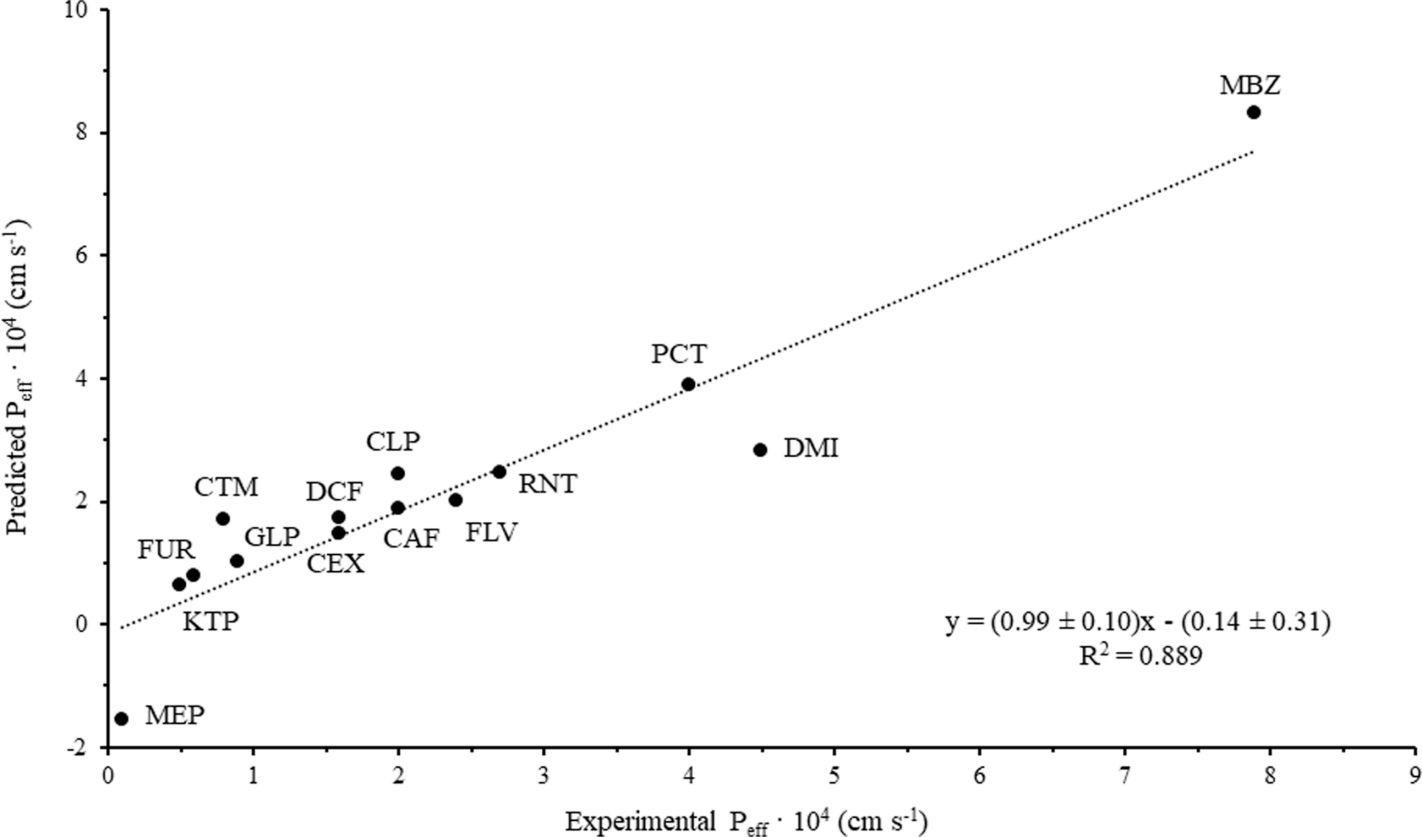
Representation of the predicted *P*_eff_ using the RMBE/LOO-based model 5 against in vivo *P*_eff_ data.

First, the model 5 was cross-validated by the leave-one-out (LOO) approach. Table 3 illustrates the absolute prediction errors for every individual compound, with values ranging from −1.69 to +0.91 cm s^–1^, thus demonstrating again that acceptable predictions are obtained with RMBE data. The low value obtained for the coefficient of variation (%) of the LOO predicted values, calculated as the sum of absolute errors divided by the sum of the in vivo *P*_eff_, is worth mentioning. Specifically, the coefficient of variation is less than 16% for aromatic compounds and only increases to 21.2% whenever nonaromatic compounds are included in the calculation. These findings demonstrate the model’s feasibility for accurately predicting effective permeability across the jejunum intestine. Then, the predicted *P*_eff_ values obtained with model 5 by the LOO technique (Table 3) were plotted against in vivo *P*_eff_ (see Figure 3) and fitted to a linear regression equation (*P*_eff (predicted)_ = (0.99 ± 0.10) *P*_eff (in vivo)_ – (−0.14 ± 0.31), *R*^2^ = 0.889). The correlation between the in vitro predicted against the in vivo *P*_eff_ was investigated using *t*-tests for comparison of the experimental values of the intercept and slope to the ideal situation of zero intercept and slope equal to 1. The statistics *t* of the slope and intercept were calculated as follows:^47^*t* = (|*b* – 1|)/*s*_b_ and *t* = (*a* – 0)/*s*_a_ in which *b* and *a* stand for the slope and intercept, respectively, and *s*_b_ and *s*_a_ stand for the standard deviation of the slope and intercept, respectively. The experimental *t* values (*t* = 0.07 and 0.45 for the slope and intercept, respectively) were in both cases below the *t*_critical_ value at the 0.05 significance level (*t* = 2.16), thereby indicating the reliability of model 5 for the in vitro prediction of *P*_eff_.

**Table 3:** Predicted *P*_eff_ by the Leave-One-Out Technique and the Absolute Prediction Error

CEC	predicted *P*_eff_ jejunum obtained by the LOO approach·10^4^ (cm/s)	absolute prediction error (cm/s)
PCT	3.87	–0.13
RNT	2.45	–0.25
CAF	1.88	–0.12
CLP	2.42	0.42
FUR	0.79	0.19
MBZ	8.30	0.40
GLP	1.02	0.12
KTP	0.62	0.12
DCF	1.73	0.13
FLV	2.00	–0.40
DMI	2.81	–1.69
CEX	1.46	–0.14
CTM	1.71	0.91
MET	–1.57	–1.67

## Conclusions

The unique in vitro analysis opportunities enabled by the novel analytical procedure so-called d-BMSPE, based on microsolid-phase extraction with biomembrane surrogates on a polymeric sorbent, to predict the effective permeability of CECs through the jejunum are in this work fully demonstrated. The SM and SEM analysis demonstrate the predominance of a PC monolayer on the PC-laden biosorbent material. The RMBE data was retrieved by reversed-phase HPLC and UV−vis spectroscopy. Notwithstanding the acceptable correlations obtained with only in vitro RMBE data against in vivo *P*_eff_, improved prediction models were built by combining the sorptive extraction data with molecular descriptors (e.g., Log *P*, Log *D* at pH 7.4, and the number of ARs). The optimized MLR model using the RMBE values along with Log *D* at pH 7.4 and the number of ARs afforded *R*^2^ = 0.883. We have also demonstrated in this work that the standard Log *P* parameter to predict bioparameters such as HOA or the related *P*_eff_ might not be appropriate inasmuch as the correlation between Log *P* or Log *D* against *P*_eff_ for the pharmaceutical organic compounds used in this study is negligible (*R*^2^ = 0.126 and *R*^2^ = 0.383 whenever MBZ is removed from the MLR model).

Further work is underway to further leverage the simplicity of d-BMSPE to predict human bioparameters both in pharmacological and toxicological studies for other compound classes of CECs.

## Supporting Information Available

Additional data and information on chemicals, analytical instrumentation, synthesis of LUVs, chromatographic assays, analytical workflow for elucidation of the conformation of the PC onto the biosorbent, and multicollinearity plot (PDF)
