# Supplementary material for: Biomimetic Dispersive Solid-Phase Microextraction: A Novel Concept for High-Throughput Estimation of Human Oral Absorption of Organic Compounds
Source: Anal Chem. 2023 Aug 24;95(35):13123–31. doi: 10.1021/acs.analchem.3c01749 (PMC10483468; doi:10.1021/acs.analchem.3c01749)
Supplement: Supplementary file 1 — ac3c01749_si_001.pdf [file ac3c01749_si_001.pdf]

Supporting information

Biomimetic dispersive solid-phase microextraction: A novel concept for high-throughput estimation of human oral absorption of organic compounds

Maria Pau García-Moll[a], Llucia García-Moll[a], Enrique Javier Carrasco-Correa[b*], Miquel Oliver[a], Ernesto Francisco Simó Alfonso[b], Manuel Miró[a*]

[a] *FI-TRACE Group, Department of Chemistry, University of the Balearic Islands, Carretera de Valldemossa, km 7.5, E-07122, Palma de Mallorca, Spain*

[b] *CLECEM Group, Department of Analytical Chemistry, University of Valencia, C/ Doctor Moliner, 50, 46100, Burjassot, Valencia, Spain*

Correspondence to:

Dr. Enrique Javier Carrasco-Correa

E-mail: enrique.carrasco@uv.es

Tel.: +34963544248

Prof. Manuel Miró

E-mail: manuel.miro@uib.es

Tel: +34 971172746

Number of Pages: 10

Number of Figures: 2

Number of Tables: 1

Table of content

Reagents and solutions (p. S3)

Synthesis and characterization of liposomes (p. S5)

HPLC analysis (p. S6)

Figure S1. Analytical workflow for elucidation of the conformation of the PC following anchorage to the monolith (p. S7)

Figure S2. Multicollinearity plot of the parameters listed in Table 1 (p. S8)

Table S1. Non-standardized coefficients of all the prediction models evaluated for the prediction of the effective permeability across the human intestine (p. S9)

References (p. S10)

Reagents and solutions

All solutions were prepared from analytical grade reagents and used without further purification. Ultrapure water (Millipore, Bedford, USA) with a resistivity ≥18.2 MΩ·cm was employed to prepare the standard solutions and constituted one of the components of the HPLC mobile phase. HPLC grade methanol (MeOH) and acetonitrile (ACN) were purchased from Fisher Scientific (Madrid, Spain). Analytical standards of paracetamol (PCT), ranitidine (RNT), caffeine (CAF), chloramphenicol (CLP), furosemide (FUR), mebendazole (MBZ), glipizide (GLP), ketoprofen (KTP), diclofenac (DCF), fluvastatin (FLV), desipramine (DMI), cephalexin (CEX), and metformin (MET) were purchased from Merck KGaA (Darmstadt, Germany) and Cimetidine (CTM) from Fisher Scientific (Madrid, Spain). Glycidyl methacrylate (GMA), lauroyl peroxide (LPO), and ethylene glycol dimethacrylate (EDMA) were purchased from Merck KGaA (Darmstadt, Germany), and 1-dodecanol and cyclohexanol from Fisher Scientific (Madrid, Spain).

Natural soybean L-α-phosphatidylcholine (PC), LIPOID S100, was purchased from LIPOID GmbH (Ludwigshafen, Germany) with a concentration of L-α-phosphatidylcholine not less than 94% and a lipid tail distribution of linoleic acid (C18:2, (9Z,12Z)-octadeca-9,12- dienoic acid) palmitic acid (C16:0, n-hexadecanoic acid) and oleic acid (C18:1, cis-9-octadecenoic acid) with percentages of *ca.* 63, 15 and 11%, respectively.

A 100 mM phosphate-buffered saline (PBS) solution was prepared by dissolving 1.2 g of potassium dihydrogen phosphate, 7.2 g of disodium phosphate, 40 g of sodium chloride and 1.0 g of potassium chloride in 500 mL of water. Working solution (10 mM PBS, pH 7.4) was prepared by a 10-fold dilution of the stock solution in water.

A 0.1 M iron (III) thiocyanate solution was prepared by dissolving 1.35 g of iron(III) chloride hexahydrate ($FeCl_3·6H_2O$) and 1.52 g of ammonium thiocyanate (NH_4SCN) in 50 mL of water. The solution was stored at room temperature.

Stock solutions of CECs were prepared at a concentration level of 1000 mg L^{-1} in MeOH (PCT, CLP, GLP, KTP, DCF, FLV), water (RNT, DMI, CEX, MET, CTM), ACN (CAF, FUR), or 50/50 (v/v) acetic acid/MeOH (MBZ). Intermediate stock solutions containing all the analytes at 100 mg L^{-1} were prepared in water. The standard solutions were stored at 4 ºC pending use. Working standard solutions for the d-BMSPE studies were prepared daily by appropriate dilution with PBS solution from the concentrated/intermediate stock solutions.

Synthesis and characterization of LUVs

The synthesis of LUVs was performed using an extruder set from Avanti Polar Lipids, Inc. (Alabaster, Alabama). This set includes the necessary laboratory material to perform the preparation of LUVs including a mini-extruder, syringes (1,000 μL), polycarbonate membranes (0.1 μm, 19 mm diameter), filter supports (10 mm diameter) and a holder/heating block.

LUVs were prepared by lipid film hydration[1] followed by extrusion for unilamellar liposome formation[2]. To this end, a soybean-PC (Sb-PC) stock solution was prepared by weighing 0.38 g of PC (LIPOID S100, average molecular weight of 787 g mol^{-1}) in chloroform in a round bottom flask. The solvent was removed in a rotary evaporator at 30 ºC under reduced pressure (290 mbar) for 2 h and protected from the light, followed by 2 h more under vacuum at room temperature to facilitate the quantitative removal of solvent traces while enabling a uniform dried lipid film on the flask bottom wall. Afterwards, the lipid film was hydrated with a given volume of 10 mM PBS (pH 7.4) at room temperature to afford a lipid concentration of ca. 100 mM Sb-PC. The aqueous PC solution was vortexed for 1 min every 5 min throughout 1 h to facilitate the complete resuspension of the PC in the aqueous saline medium. The milky suspension of multilamellar vesicles (MLV) was stored at 4 ºC overnight for stabilization. The LUVs were formed by extruding the MLV containing solution through a 100 nm pore size polycarbonate filter[3], which operational procedure was repeated 29 times. The characterization of LUVs was performed by dynamic light scattering (DLS) using a Zetasizer Nano ZS90 system (Malvern Panalytical, Malvern, UK). The evaluation of the anchored LUVs onto the porous organic polymer surfaces was performed by UV-Vis spectrophotometry (UV-Vis Carry 300 Bio) and scanning electron microscopy (SEM) (HITACHI S-3400N).

Liquid chromatographic analysis

Chromatographic analyses were performed on a LC-4000 HPLC system (Jasco, Tokyo, Japan) equipped with a high-pressure quaternary pump (PU- 4180), a photodiode array detector (MD-4017). The separation of the analytes was performed on an Onyx Monolithic HD-C18 LC Column (250 × 4.6 mm). The HPLC system was operated under gradient mode using a mobile phase consisting of (A) H_2O + 0.1% of HAcO, with a gradient percentage from 95 to 50 in 26 min, and (B) ACN, from 5 to 50%. The flow rate throughout the monolithic column was 2 $mL\cdot min^{-1}$, the column temperature was set at 30 ºC, and the injection volume was 20 μL. The analytes were measured at their maximum detection wavelength in the range of 215-354 nm, except MET. The highly polar MET overlapped with the injection peak by reversed phase chromatography and thus direct UV detection at 233 nm was performed instead.

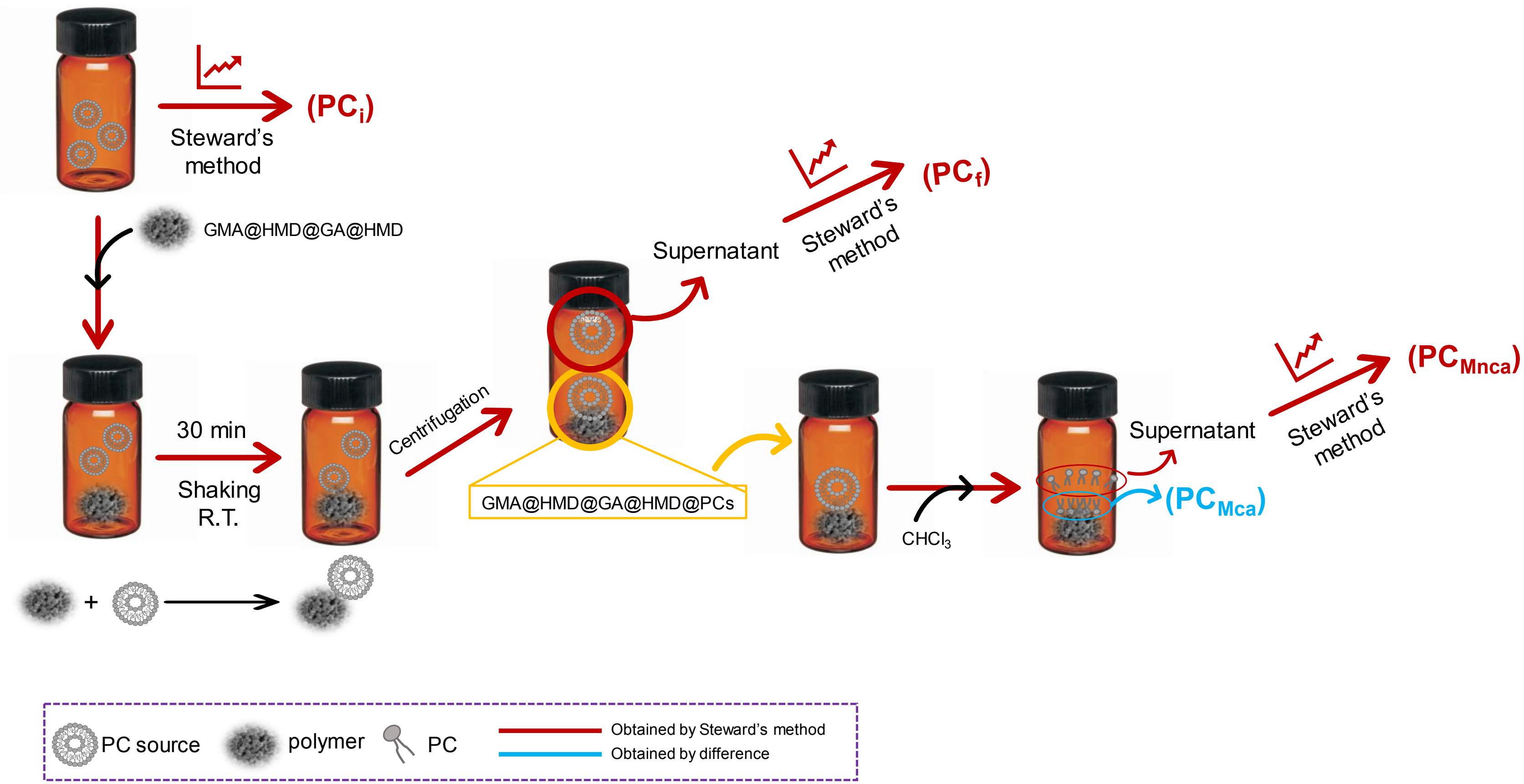

Figure S1. Analytical workflow for elucidation of the conformation of the PC following anchorage to the monolith (monolayer, bilayer or vesicles). PC$_i$, PC$_f$ and PCM stand for the initial PC before anchoring, the PC that was not anchored to the polymer and the PC anchored to the polymer after reaction between the powder ground monolith material and the LUVs, respectively. PC$_{Mca}$ and PC$_{Mnca}$ stand for PC covalently attached to the polymer and PC not covalently attached but bound to the polymer, respectively. LUV image was selected as example of PC conformation onto the polymer surface.

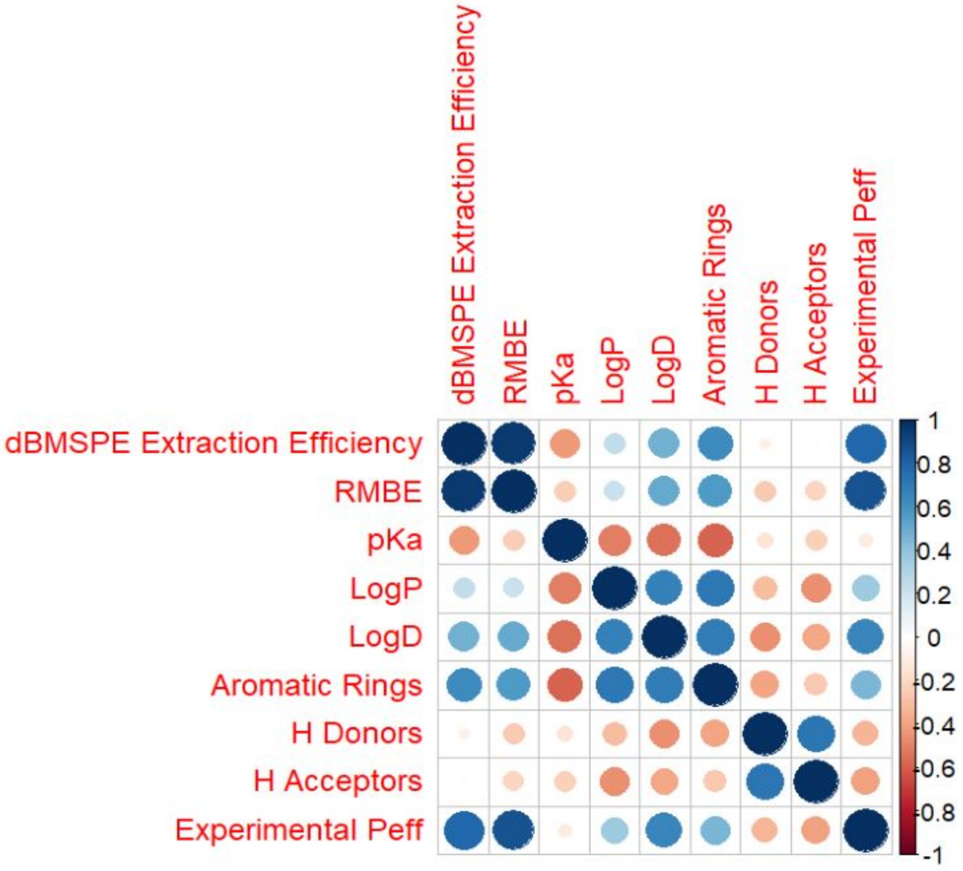

Figure S2. Multicollinearity plot of the parameters listed in Table 1.

Table S1. Non-standardized coefficients of all the prediction models evaluated for the prediction of the effective permeability across the human intestine

model	Constant parameter	RMBE·10^4 (mol CEC/mol PC)	pK_a	Log P	Log D (7.4)	Number aromatic rings	Number H-donor	Number H-acceptor	R^2
1	1.71	-	-	0.362	-	-	-	-	0.1264
2	2.29	-	-	-	0.649	-	-	-	0.4329
2*	1.94	-	-	-	0.377	-	-	-	0.3035
**3**	**0.51**	**0.040**	**-**	**-**	**-**	**-**	**-**	**-**	**0.7628**
3*	0.56	0.038	-	-	-	-	-	-	0.3826
**4**	**0.82**	**0.033**	**-**	**-**	**0.288**	**-**	**-**	**-**	**0.8266**
4*	0.86	0.032	-	-	0.288	-	-	-	0.5489
**5**	**2.05**	**0.038**	**-**	**-**	**0.477**	**-0.864**	**-**	**-**	**0.8828**
5*	2.01	0.040	-	-	0.479	-0.874	-	-	0.6956
**6**	**-0.14**	**0.041**	**0.088**	**-**	**-**	**-**	**-**	**-**	**0.7729**
**7**	**0.33**	**0.038**	**-**	**0.169**	**-**	**-**	**-**	**-**	**0.7888**
**8**	**0.69**	**0.041**	**-**	**-**	**-**	**-0.147**	**-**	**-**	**0.7652**
**9**	**1.17**	**0.038**	**-**	**-**	**-**	**-**	**-0.261**	**-**	**0.7799**
**10**	**2.17**	**0.038**	**-**	**-**	**-**	**-**	**-**	**-0.385**	**0.8160**
**11**	**0.97**	**0.033**	**-**	**-**	**0.273**	**-**	**-0.067**	**-**	**0.8276**
11*	1.10	0.031	-	-	0.268	-	-0.087	-	0.5526
**12**	**1.94**	**0.033**	**-**	**-**	**0.226**	**-**	**-**	**-0.276**	**0.8510**
12*	2.45	0.025	-	-	0.211	-	-	-0.339	0.6299
**13**	**2.38**	**0.038**	**-**	**-**	**0.453**	**-0.890**	**-0.130**	**-**	**0.8863**
**13***	**2.38**	**0.038**	**-**	**-**	**0.452**	**-0.890**	**-0.130**	**-**	**0.7039**
**14**	**3.15**	**0.038**	**-**	**-**	**0.415**	**-0.859**	**-**	**-0.272**	**0.9065**
**14***	**3.40**	**0.038**	**-**	**-**	**0.400**	**-0.832**	**-**	**-0.308**	**0.7621**

* Model obtained without MBZ

Coefficients in bold for models with R^2>0.7

References

1. Bangham, A. D., de Gier, J., & Greville, G. D. (1967). Osmotic properties and water permeability of phospholipid liquid crystals. *Chem. Phys. Lipids* 1, 225-246. https://doi.org/10.1016/0009-3084(67)90030-8
2. Hope, M. J., Bally, M. B., Webb, G., & Cullis, P. R. (1985). Production of large unilamellar vesicles by a rapid extrusion procedure. Characterization of size distribution, trapped volume and ability to maintain a membrane potential. *BBA - Biomembranes* 812, 55-65. https://doi.org/10.1016/0005-2736(85)90521-8
3. Li, M., Du, C., Guo, N., Teng, Y., Meng, X., Sun, H., Li, S., Yu, P., & Galons, H. (2019). Composition design and medical application of liposomes. *Eur. J. Med. Chem.* 164, 640-653. https://doi.org/10.1016/j.ejmech.2019.01.007
